# The Protective Mechanism of CAY10683 on Intestinal Mucosal Barrier in Acute Liver Failure through LPS/TLR4/MyD88 Pathway

**DOI:** 10.1155/2018/7859601

**Published:** 2018-03-13

**Authors:** Yao Wang, Hui Chen, Qian Chen, Fang-Zhou Jiao, Wen-Bin Zhang, Zuo-Jiong Gong

**Affiliations:** ^1^Department of Infectious Diseases, Renmin Hospital of Wuhan University, Wuhan, China; ^2^Hubei Provincial Center for Disease Control and Prevention, Wuhan, China

## Abstract

The purpose of this study was to investigate the protective mechanism of HDAC2 inhibitor CAY10683 on intestinal mucosal barrier in acute liver failure (ALF). In order to establish ALF-induced intestinal epithelial barrier disruption models, D-galactosamine/LPS and LPS were, respectively, used with rats and NCM460 cell and then administrated with CAY10683. Transepithelial electrical resistance (TEER) was measured to detect the permeability of cells. Real-time PCR and Western blotting were employed to detect the key mRNA and protein levels. The intestinal epithelial tissue pathology was detected. After interfering with CAY10683, the mRNA and protein levels of TLR4, MyD88, TRIF, and TRAF6 were decreased compared with model group (*P* < 0.05), whereas the levels of ZO-1 and occluding were elevated (*P* < 0.05). The permeability was elevated in CAY10683-interfered groups, when compared with model group (*P* < 0.05). And the degree of intestinal epithelial tissue pathological damage in CAY10683 group was significantly reduced. Moreover, CAY10683 significantly decreased the TLR4 staining in animal tissue. The HDAC2 inhibitor CAY10683 could promote the damage of intestinal mucosal barrier in ALF through inhibiting LPS/TLR4/MyD88 pathway.

## 1. Introduction

Acute liver failure (ALF), caused by a large area of hepatocyte necrosis, is a serious clinical syndrome and characterized by the rapid progress of hepatic encephalopathy and severe liver damage associated with high mortality [1]. Early monitor of gastrointestinal dysfunction is a key process to the development of ALF. Endotoxin is a lipopolysaccharide component located in gram-negative bacterial cell wall that easily passes through the damaged intestinal mucosal barrier. Under normal circumstances, the intestinal tract contains a large number of potential pathogens and endotoxin, but the intact intestinal mucosal barrier can effectively prevent intestinal bacteria and endotoxin into the body [2].

Systemic and intestinal immune system in the early stage of ALF showed the inhibited state, so the removal of bacterial capacity decreased and the displacement of bacteria could easily break through the limitations of mesenteric lymph nodes to migrate and colonize to the other body tissues and organs, which could result intestinal mucosal barrier dysfunction, increased permeability, intestinal bacterial translocation, and intestinal endotoxemia, and the result in inflammatory response can further cause systemic inflammatory response syndrome (SIRS) and multiple organ dysfunction syndrome (MODS) [3]. Intestinal *Escherichia coli* and endotoxin penetrate the intestinal epithelium into the blood circulation, activating the mononuclear macrophage system, to promote the release of a large number of cell “toxic factors,” such as cytokines, inflammatory mediators, proteases, and oxygen-free radicals [4]. On the one hand, LPS could aggravate the inflammatory injury of the intestinal mucosal barrier, promote the migration of bacteria and endotoxin, and thus format a vicious cycle. On the other hand, the cytokines invading into the blood circulation could result in persistent inflammatory response and continued self-enhancement. Interaction between LPS and TLR4 leads to formation of an LPS signaling complex consisting of surface molecules, including myeloid differentiation primary response gene 88 (MyD88), toll-interleukin-1 receptor domain-containing adaptor inducing interferon *β* (TRIF), TNF-*α* receptor association factor 6 (TRAF6), and activation of transcription factors, which then induce the activation of the inflammatory response [5]. The formation of cascade reaction constitutes a “second strike” to promote systemic inflammatory response syndrome and the occurrence of multiple organ dysfunction [6]. Therefore, the protective of intestinal mucosal barrier can effectively alleviate the ALF.

Aberrant expression of HDAC2 has been identified in chronically inflamed tissues [7]. In human cervical cancer cell lines, HDAC2 was reported to inhibit transcription of the expression of MHC class II genes, and antigen presentation through MHC class II is critical for activation of adaptive immune responses [8]. Cluster of differentiation 36 (CD36) could aggravate macrophage infiltration and hepatic inflammation by upregulating monocyte chemotactic protein-1 (MCP-1) expression of hepatocytes through upregulating HDAC2-dependent pathway [9]. Therefore, histone deacetylase inhibitor (HDACi) may be used to modify immunity through multiple hosts to improve the efficacy of anti-inflammation therapy. Santacruzamate A (CAY10683) is a potent selective HDAC inhibitor that has an IC50 of 119 pM for HDAC2 and a >3600-fold selectivity over other HDACs [10]. As a clinically approved HDAC inhibitor, CAY10683 has antiproliferative and immunomodulatory effects [11], thereby CAY10683 was used to treat cutaneous T-cell lymphoma [12] and breast cancer [13].

Our previous studies have indicated that the selective class I and II HDAC inhibitor trichostatin A could protect the small intestine in ALF liver failure by inflammatory inhibition [6]. However, it is still unknown which type of HDAC molecule inhibitor could protect the small intestine through which kind of specific molecular mechanism. In this study, we aimed to investigate the protective effect of selective HDAC2 inhibitor CAY10683 on LPS-induced damage in NCM460 cells line and in galactosamine/LPS-induced ALF rat model and to explore the mechanisms.

## 2. Material and Methods

### 2.1. Chemicals and Reagents

CAY10683 was purchased from Sellect (Houston, USA). DMEM basic and fetal bovine serum (FBS) were purchased from Gibco (NY, USA). Lipopolysaccharide (LPS, purity of 99%) and D-galactosamine (purity of 98%) were purchased from Sigma (St. Louis, USA). The cell counting kit-8 (CCK-8) was purchased from Dojindo Laboratories (Japan). Rabbit anti-rat/human HDAC2, histone 3, acetyl-histone 3 (Ac-histone 3), MyD88, TRIF, TRAF6, occluding, and *β*-actin were obtained from Cell Signaling Technology (CST) (Boston, USA). TLR4 and ZO-1 antibodies were purchased from Proteintech (Wuhan, China). The goat anti-rabbit fluorescent secondary antibody (IRDye800) was obtained from LI-COR Biosciences Inc. (Lincoln, USA). RNAiso Plus, PrimeScript™ RT reagent kit, and SYBR Premix Ex Taq kit were purchased from TaKaRa (Dalian, China).

### 2.2. Cell Culture and Chemical Treatment

DMEM medium mixed with 10% FBS was used to culture NCW460 cells in an incubator at 37°C, 5% CO_2_, and saturated humidity [14]. The medium was replaced every 2-3 days. LPS was used for cellular model establishment. The intervention groups were divided into normal group, model group, and CAY 10683 group. After the cells were passed in 6-well plates (for real-time PCR and Western blot) or 96-well plates (for CCK8 experiments) for 24 h and cultured to 70% density, the supernatants were removed and LPS (1 *μ*g/ml) was used to stimulate the cells excluding the normal group. At the last 12 hours, CAY 10683 (120 nM) was added into the wells expected for the normal group and model group. After 24 h, the cells were harvested.

### 2.3. Cell Viability Assays

The cell counting kit-8 (CCK-8) assay was used to examine cell proliferation. NCM460 cells were seeded in 96-well plates at a density of 1.0 × 10^5^/ml for 24 h. After incubation, the medium was replaced with 100 *μ*l various concentrations of CAY 10683 (10 nM, 100 nM, 1000 nM, 10^4^ nM, 10^5^ nM, 10^6^ nM, 10^7^ nM, and 10^8^ nM). DMEM medium substituted with 10 *μ*l CCK-8 solution was added 24 h later, and the cells were incubated at 37°C. After 2 h, the absorbance at 490 nm was read on microplate reader.

### 2.4. Transepithelial Electrical Resistance (TEER) Measurement

NCM460 cells were made into 2.5 × 10^5^ cells/ml single cell suspension. The lower cell chambers were added with 1.5 ml DMEM complete medium, and the upper cell chambers were added with 1 ml cell suspension, incubated overnight in an incubator at 37°C, 5% CO_2_, and saturated humidity. To observe whether the cell formed a tight layer, and there were no holes formed by dead cells, if the conditions were not met, the cells should be recultured. According to the experimental groups, upper and lower chambers in the model and CAY10683 groups were replaced by the complete medium and complete medium included CAY10683. After 2 hours, the CAY10683 group and LPS group at the same time were added by LPS. The normal group was added by complete medium as control. The cells were incubated for 24 h. And then the calibrated Millipore Millicell ERS-2 cell resistance meter was used to detect resistance value. The measured resistance value was multiplied by the area of the filter to obtain an absolute value of TEER, expressed as Ω cm^2^. And the TEER values were measured as follows: TEER = (measured resistance value−blank value) × single cell layer surface area (cm^2^).

### 2.5. Animal Groups

Followed the previously published steps [2], a total of 18 rats were randomly divided into three groups with six rats in each group: normal, model, and CAY10683 group. The ALF rat models were administrated by intraperitoneal injection with 400 mg/kg D-galactosamine combined with 100 *μ*g/kg of LPS. CAY10683 and the same amount of saline, respectively, were given to the CAY10683 group and model group 2 h before ALF model protocol was conducted. All animals were killed in 48 h.

### 2.6. Biochemical Tests

The serum alanine aminotransferase (ALT), aspartate aminotransferase (AST), and total bilirubin (TBIL) levels were assayed using a fully automated Aeroset chemistry analyzer provided by Abbott Co. Ltd.

### 2.7. Specimen Collection and Histological Studies

The procedures of the H&E staining assays followed the previously described steps [15]. Fresh liver and small intestine specimens were fixed in 10% neutral-buffered formalin for 2 days and then processed for sectioning and staining by standard histological methods. Sections from the liver and small intestine were stained with H&E and evaluated under BX 51 light microscope (Olympus, Japan).

### 2.8. Quantitative Real-Time PCR to Detect mRNA Expression

Total RNA in NCM460 cells and small intestine specimens was isolated by using RNAiso Plus according to manufacturer's protocol. The cDNAs were produced with a PrimeScript RT reagent kit and incubated at 37°C for 15 min and at 85°C for 5 s. Real-time PCRs were performed using a StepOne Plus device (Applied Biosystems) at 95°C for 10 s, followed by 40 cycles at 95°C for 5 s, and at 60°C for 20 s, according to the instructions for the SYBR Premix Ex Taq kit. The data were analyzed by the 2^−ΔΔCT^ method. All the primers were synthesized by Tsingke (Wuhan, China), and the sequences are listed in Table 1.

### 2.9. Western Blotting for Protein Expression Measurement

Western blots were carried out using whole cell and small intestine specimen extracts separated on SDS-PAGE gels and then transferred onto a nitrocellulose filter membrane. The membranes were blocked overnight with 5% nonfat milk in phosphate-buffered saline (PBS) and probed with the indicated antibody (Ab) before being washed three times in Tris-buffered saline with Tween 20 (TBST) and then incubated with an HRP-labeled secondary Ab. The dilutions of the primary and secondary antibodies were as follows: HDAC2, 1 : 1000; TLR4, 1 : 1000; MyD88, 1 : 1000; TRIF, 1 : 1000; TRAF6, 1 : 1000; ZO-1, 1 : 1000; occluding, 1 : 1000; histone 3, 1 : 1000; acetyl-histone 3, 1 : 1000; and *β*-actin, 1 : 1000. And then this was followed by incubation with a fluorescent secondary antibody at 37°C for 2 h. The blot was analyzed using the Odyssey Infrared Imaging System (LI-COR Biosciences). Membranes were also probed for *β*-actin and histone 3 as additional loading controls.

### 2.10. IHC to Detect TLR4 Expression in Liver Tissue

After dewaxing and hydrating, the small intestine specimens were cut into 5 mm sections and then incubated in 3% H_2_O_2_ methanol to eliminate endogenous peroxidase activity. The sections were incubated with normal goat serum for 10 min and incubated with TLR4 (1 : 200) antibody overnight at 4°C. Horseradish peroxidase- (HRP-) conjugated polyclonal to rabbit IgG was used to incubate the sections at 37°C for 30 min after being rinsed again with PBS. The samples were developed with diaminobenzidine and stained with hematoxylin. At last, Image-Pro Plus 6.0 (IPP) software was used to analyze the optical density of the images.

### 2.11. Intestinal Permeability

Small intestinal mucosal barrier function was assessed using an everted sac method as our previously described [6]. Segments were everted in ice-cold Krebs buffer (pH 7.4), gently distended by injecting 1.5 ml of Krebs, and then suspended in the organ bath for 30 min. The organ bath contained 500-ml Krebs with added FITC-labeled dextran 4000 (FD4, 10 mg/ml) and maintained at 37°C, continuously bubbled with a gas mixture containing 95% O_2_ and 5% CO_2_. Samples from the sac were centrifuged at 1000*g* at 4°C for 5 min. FD4 concentration was detected at an excitation wavelength of 492 nm and an emission wavelength of 515 nm with PerkinElmer LS-50 fluorescence spectrophotometer (PerkinElmer Inc., Waltham, MA). Intestinal permeability was expressed as FD4 concentration divided by the area of gut sac.

### 2.12. Statistical Analysis

All statistical analyses were performed with SPSS 12.0. The results were expressed as means ± SDs. Results were performed with Student's *t*-test and one-way analysis of variance (ANOVA). *P* < 0.05 was considered statistically significant.

## 3. Results

### 3.1. Detection of Cell Proliferation Activity by CCK-8

**Figure 1:**
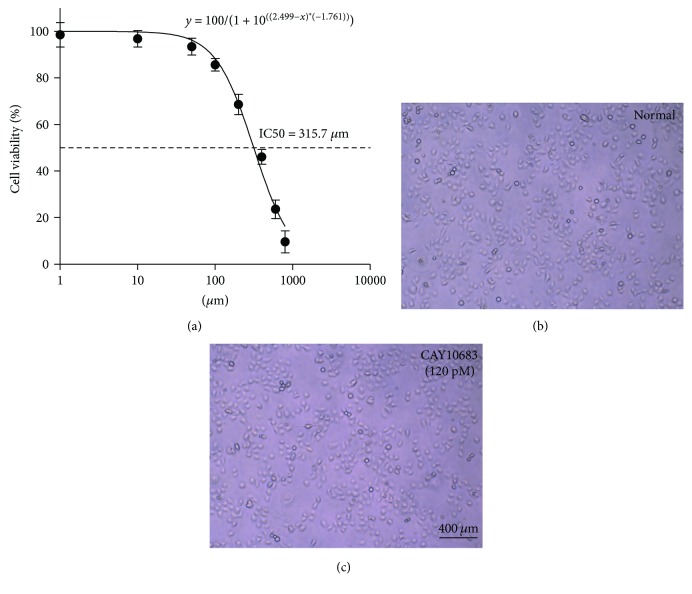
(a) Detection of cell proliferation activity by CCK-8. (b–c) Cell morphology was observed in normal and CAY10683-treated NCM460 cell group. Values are expressed as mean ± SD. *P* < 0.05, compared with normal group; *P* < 0.05, compared with model group.

Cell viability (%) was calculated as follows: [A (experimental well)−A (blank well)]/[A (control well)−A (blank well)] × 100. Moreover, we assessed the viability of cells treated with different concentrations (10 nM, 100 nM, 1000 nM, 10^4^ nM, 10^5^ nM, 10^6^ nM, 10^7^ nM, and 10^8^ nM) of CAY10683 on after 24 h. Based on the CCK8 assay, the concentration-effect curve was represented by the equation *y* = 100/(1 + 10^((2.499−*x*)^^∗^^(−1.761))^) (Figure 1(a)), and the specific parameters IC50 (315.7 nM), hillslope (−1.761), and *R*^2^ (0.9748) were also assessed. Against HDAC2, CAY10683 showed an inhibitory concentration of 119 pM, which is over 3600-fold more potent than other HDACs [10]. For convenience, we approximated the concentration 120 pM and the viability was 99.99% for NCM460. Thus, the pretreatment concentrations of CAY10683 of unstimulated NCW460 cells were 120 pM for 24 h, none of which significantly affected cell morphology (Figures 1(b) and 1(c)). Therefore, we chose to treat cells with CAY10683 at 120 pM for 24 h in this experiment.

### 3.2. Effect of CAY10683 on TEER in LPS-Stimulated NCM460 Cells

**Figure 2:**
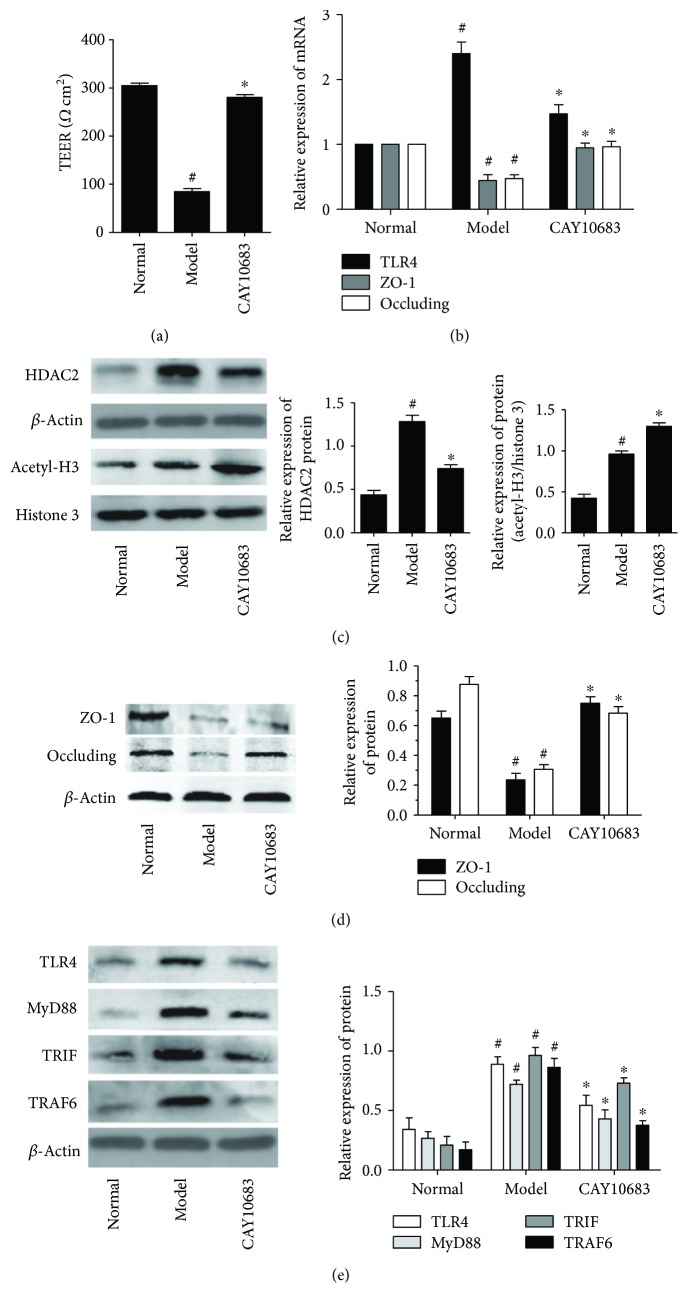
(a) Effect of CAY10683 on TEER in LPS-stimulated NCM460 cells. (b) Effect of CAY10683 on the mRNA expression of TLR4, ZO-1, and occludin in LPS-stimulated NCM460 cell. (c) Effect of CAY10683 on HDAC2 and acetyl-H3 in LPS-stimulated NCM460 cells. (d) Effect of CAY10683 on the protein levels of ZO-1 and occludin in LPS-stimulated NCM460 cell. (e) Effect of CAY10683 on the TLR4, MyD88, TRIF, and TRAF6 protein levels in LPS-stimulated NCM460 cells. Values are expressed as mean ± SD. ^#^*P* < 0.05, compared with normal group; ^∗^*P* < 0.05, compared with model group.

As the TEER became higher, the cell permeability was lower. The small chamber film diameter, aperture, and surface area were 12 mm, 0.4 *μ*m, and 1.13 cm^2^. The background resistance of blank was deducted from each value. As shown in Figure 2(a), compared with the normal group, the TEER value in the model group was significantly decreased (*P* = 0.000, fold change (FC) = 0.28). After being treated with CAY10683, the TEER value was pronouncedly elevated (*P* = 0.000, FC = 3.31).

### 3.3. Effect of CAY10683 on Acetylation Regulation in LPS-Stimulated NCM460 Cells

CAY10683 is a HDACi that specifically targets the HDAC2 and consequently affects the acetylation of histone. To confirm the effects of CAY10683 on HDAC2 and histone, we firstly verify the effect of CAY10683 on HDAC2 and acetylation of histone H3. Stimulated by LPS, the expression of HDAC2 was increased in NCM460 cells (*P* = 0.001, FC = 2.94). As expected, CAY10683 showed inhibition on HDAC2 in cells (Figure 2(c), *P* = 0.003, FC = 0.577). The acetylated histone H3 was also promoted by LPS simulation (*P* = 0.001, FC = 2.27) and then enhanced by CAY10683 (Figure 2(c), *P* = 0.005, FC = 1.35).

### 3.4. Effect of CAY10683 on the mRNA and Protein Expression of ZO-1 and Occludin in LPS-Stimulated NCM460 Cell

As shown in Figures 2(b) and 2(d), compared with the normal group, the ZO-1 (mRNA: *P* = 0.004, FC = 0.473; protein: *P* = 0.003, FC = 0.364) and occluding (mRNA: *P* = 0.005, FC = 0.51; protein: *P* = 0.001, FC = 0.350) mRNA and protein levels in the model group were significantly decreased. After being treated with CAY10683, the expression of ZO-1(mRNA: *P* = 0.013, FC = 1.95; protein: *P* = 0.001, FC = 3.17) and occluding (mRNA: *P* = 0.014, FC = 1.82; protein: *P* = 0.002, FC = 2.23) was pronouncedly elevated.

### 3.5. Effect of CAY10683 on the TLR4/MyD88 Pathway in LPS-Stimulated NCM460 Cells

**Figure 3:**
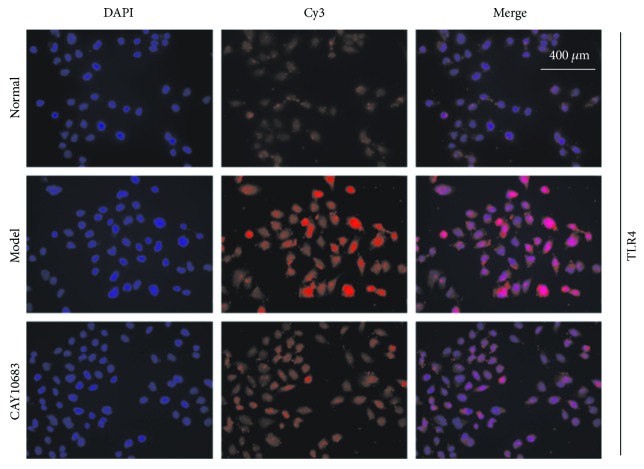
The IF method was used to detect the protein expressions of TLR4 in LPS-stimulated NCM460 cell.

Next, we evaluated the effect of CAY10683 on the TLR4/MyD88 pathway in LPS-stimulated NCM460 cells. The TLR4 mRNA (*P* = 0.000, FC = 2.40) and TLR4 (*P* = 0.001, FC = 2.62), MyD88 (*P* = 0.001, FC = 2.70), TRIF (*P* = 0.001, FC = 3.19), and TRAF6 (*P* = 0.001, FC = 3.07) protein levels were dramatically increased after LPS administration. On the contrary, CAY10683 administration appeared to downregulate the TLR4 mRNA (*P* = 0.002, FC = 0.613) and TLR4 (*P* = 0.005, FC = 0.610), MyD88 (*P* = 0.004, FC = 0.597), TRIF (*P* = 0.032, FC = 0.714), and TRAF6 (*P* = 0.002, FC = 0.454) protein levels (Figures 2(b) and 2(e)). Similarly, we furtherly used immunofluorescence (IF) method to detect the protein expressions of TLR4 in LPS-stimulated NCM460 cell. The TLR4 protein level in the model group was significantly increased (*P* = 0.001, FC = 2.43), and CAY10683 could notably decrease TLR4 protein expression (Figure 3, *P* = 0.002, FC = 0.632).

### 3.6. Effect of CAY10683 on Hepatic Pathological Changes and Serum Biochemical Indicators in ALF Rats

**Figure 4:**
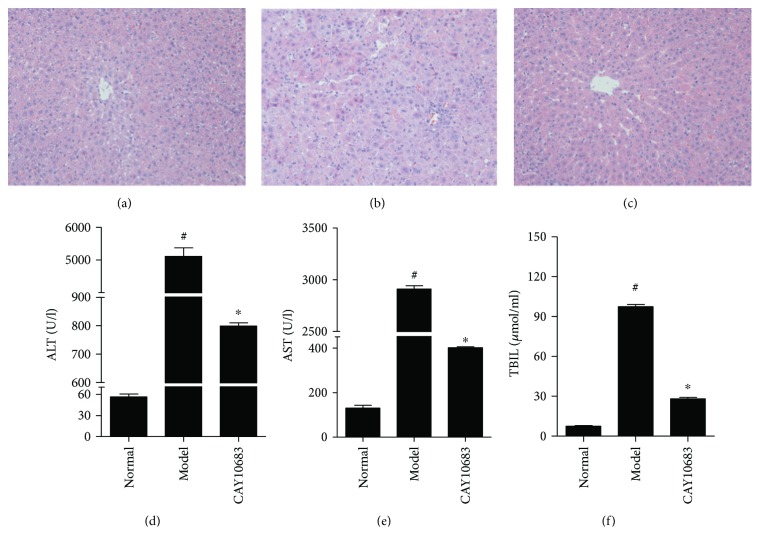
Effect of CAY10683 on hepatic pathological changes and serum biochemical indicators in ALF rats: (a) normal group, (b) model group, and (c) CAY10683 group. (d–f) Effect of CAY10683 on serum ALT, AST, and TBIL in ALF rats. Values are expressed as mean ± SD. ^#^*P* < 0.05, compared with normal group; ^∗^*P* < 0.05, compared with model group.

Because the liver is the main target organ in ALF caused by LPS, both histology and function are certainly injured to some extent. Therefore, in vivo experiment, we primarily detect whether the ALF rats model was successfully established and then detect the effect of CAY10683 on liver pathological changes and serum biochemical indicators in ALF rats. As H&E staining shown in Figures 4(a)–4(c), the structure of liver lobules in the normal group was clear, the arrangement of liver cells was neat, and the infiltration of inflammatory cells was not observed around the liver cells. The liver lobular structure in the ALF model group was unclear, and hepatocytes were necrotic surrounded by inflammatory cell infiltration. The hepatic lobule structure in CAY10683 rat liver was clearer than that in the ALF model group, and the infiltration of inflammatory cells was also reduced. As shown in Figures 4(d)–4(f), the serum ALT (*P* = 0.000, FC = 91.3), AST (*P* = 0.000, FC = 22.3), and TBIL (*P* = 0.000, FC = 13.2) in the model group were in the higher levels. Compared with the model group, the CAY10683-treated groups showed significant decreases in the ALT (*P* = 0.000, FC = 0.156), AST (*P* = 0.000, FC = 0.138), and TBIL (*P* = 0.000, FC = 0.287) levels.

### 3.7. Effect of CAY10683 on Small Intestine Damages in ALF Rats

**Figure 5:**
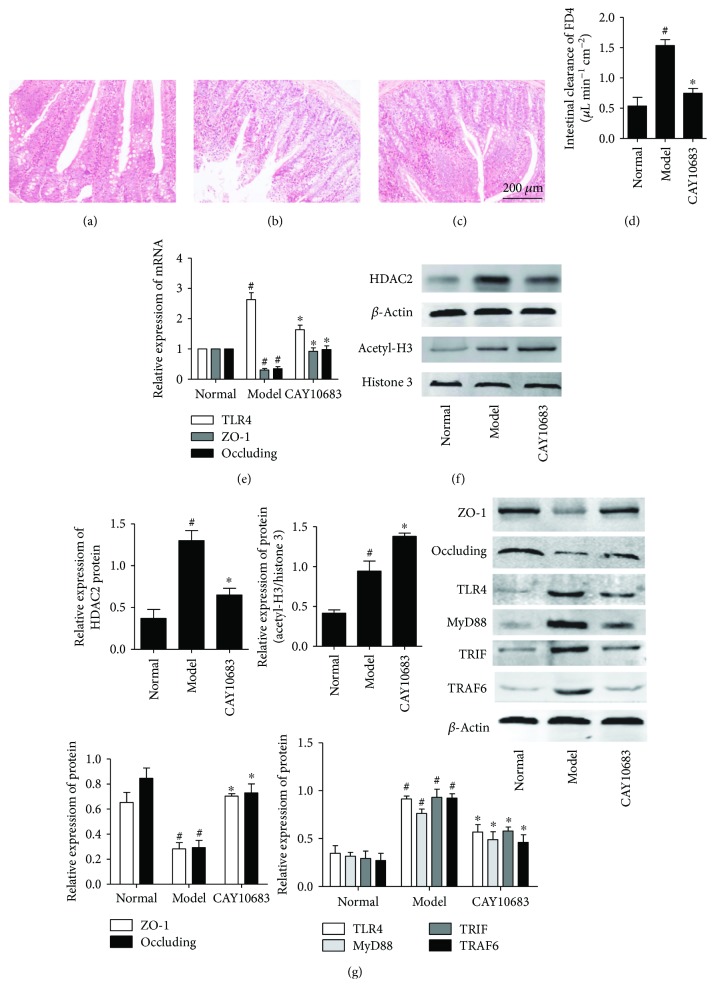
(a–c) Effect of CAY10683 on small intestine damages in ALF rats: (a) normal group, (b) model group, and (c) CAY10683 group. (d) Effect of CAY10683 on the intestinal permeability in ALF rats. (e) Effect of CAY10683 on the mRNA expression of TLR4, ZO-1, and occludin in ALF rats. (f) Effect of CAY10683 on HDAC2 and acetyl-H3 in ALF rats. (g) Effect of CAY10683 on the protein levels of ZO-1, occludin, TLR4, MyD88, TRIF, and TRAF6 protein levels in ALF rats. Values are expressed as mean ± SD. ^#^*P* < 0.05, compared with normal group; ^∗^*P* < 0.05, compared with model group.

We assessed the histology and intestinal permeability changes of jejunum (5 cm away from pylorus) (Figures 5(a)–5(c)). Histologic analysis revealed mucosal damage in the model group, with elevation of the epithelial layer from the lamina propria, denuding and loss of height of the villi, and large reactive lymphoid follicles along with excess of lymphocytes in lamina propria. However, the effects of ischemia and reperfusion were markedly alleviated by CAY10683. Along with the changes in histology, the intestinal permeability significantly increased in the model group compared with the control (*P* = 0.001, FC = 2.85) and was dramatically improved by CAY10683 (*P* = 0.000, FC = 0.488, Figure 5(d)).

### 3.8. Effect of CAY10683 on Acetylation Regulation in ALF Rats

Compared with the normal group, the expression of HDAC2 was increased in the model group (*P* = 0.001, FC = 3.51). CAY10683 showed inhibition on HDAC2 ALF rats (Figure 5(f), *P* = 0.001, FC = 0.500). The acetylated histone H3 was also promoted in the model group (*P* = 0.002, FC = 2.26) and then enhanced by CAY10683 (Figure 5(f), *P* = 0.005, FC = 1.46).

### 3.9. Effect of CAY10683 on the mRNA and Protein Expression of ZO-1 and Occludin in ALF Rats

As shown in Figures 5(e) and 5(g), compared with the normal group, the ZO-1 (mRNA: *P* = 0.000, FC = 0.307; protein: *P* = 0.002, FC = 0.434) and occluding (mRNA: *P* = 0.000, FC = 0.35; protein: *P* = 0.001, FC = 0.346) mRNA and protein levels in the model group were significantly decreased. After being treated with CAY10683, the expression of ZO-1 (mRNA: *P* = 0.001, FC = 3.01; protein: *P* = 0.000, FC = 2.48) and occluding (mRNA: *P* = 0.002, FC = 2.80; protein: *P* = 0.001, FC = 2.49) were pronouncedly elevated.

### 3.10. Effect of CAY10683 on the TLR4/MyD88 Pathway in ALF Rats

**Figure 6:**
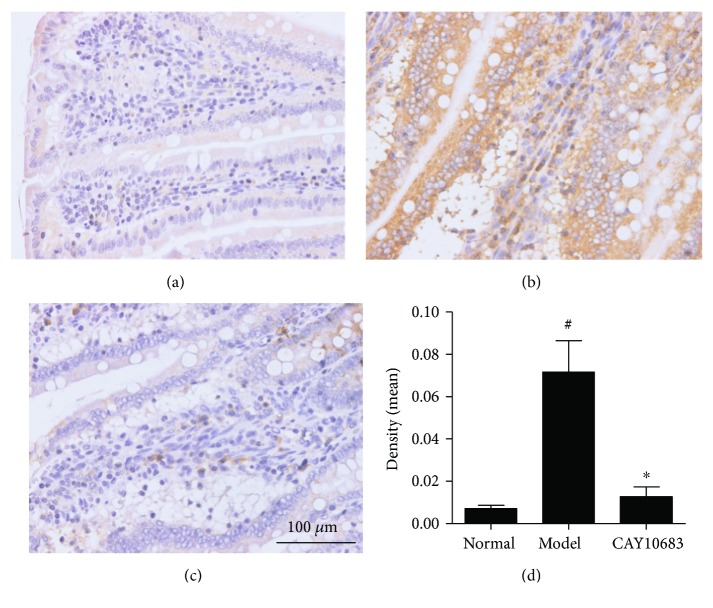
(a–d) Detection of TLR4 protein level in liver tissues with IHC method: (a) normal group, (b) model group, and (c) CAY10683 group. Values are expressed as mean ± SD. ^#^*P* < 0.05, compared with normal group; ^∗^*P* < 0.05, compared with model group.

Finally, we evaluated the effect of CAY10683 on the TLR4/MyD88 pathway in ALF rats. The TLR4 mRNA (*P* = 0.000, FC = 2.63) and TLR4 (*P* = 0.000, FC = 2.63), MyD88 (*P* = 0.000, FC = 2.41), TRIF (*P* = 0.001, FC = 3.17), and TRAF6 (*P* = 0.000, FC = 3.42) protein levels were dramatically increased after modeling. On the contrary, CAY10683 administration appeared to downregulate the TLR4 mRNA (*P* = 0.003, FC = 0.622) and TLR4 (*P* = 0.002, FC = 0.624), MyD88 (*P* = 0.007, FC = 0.642), TRIF (*P* = 0.003, FC = 0.624), and TRAF6 (*P* = 0.001, FC = 0.498) protein levels (Figures 5(e) and 5(g)). The IHC method was used to detect the protein expressions of TLR4 in liver tissues, which mainly expressed in cell membranes and cytoplasm. The TLR4 protein level in the model group was significantly increased (*P* = 0.002, FC = 10.12) and CAY10683 could notably decrease TLR4 protein expression (Figures 6(a)–6(d), *P* = 0.003, FC = 0.178).

## 4. Discussion

Intestinal mucosa barrier dysfunction can cause intestinal flora disturbance in ALF, and gram-negative bacilli reproduce excessively, producing a large amount of endotoxin [16]. However, the damaged liver cannot completely clear endotoxin, which not only leaded to excessive endotoxin get into the systemic circulation, but also exacerbated the endotoxin damage to the liver [17]. Endotoxin induced the production of inflammatory cytokines, destruction of intestinal mucosal barrier function, resulting in increased intestinal mucosal permeability.

Intestinal mechanical barrier is mainly composed of intestinal mucosal epithelial cells and intercellular connection [18]. And the intestinal mechanical barrier transmembrane proteins ZO-1 and occludin are important structures that make up a tight junction, which determines the permeability of the intestinal barrier and intestinal tract [19]. Once the transmembrane proteins are reduced, missed, or mutated, the intestinal epithelial cell permeability would increase [20], and bacteria, lipopolysaccharides, and macromolecules would enter the systemic circulation through tight junctions [21, 22], resulting in local or systemic inflammatory responses that can lead to MODS, even caused death [23]. In addition, LPS can directly destroy the tight junction protein, leading to decreased resistance to intestinal epithelial [24]. At present, LPS mainly through the role of cell membrane surface receptors such as TLR4 activates the MyD88 downstream signaling pathway, thus affecting the expression of tight junction proteins [25–27]. TLR-4 recognizes pathogens, then passes the activation signals into cells, upregulates transcription factors such as MyD88, promotes the release of cytokines or inflammatory mediators, activates local or systemic inflammatory responses, causes structural changes, or decreases the expression of tight junction proteins, finally leading to intestinal mucosal barrier dysfunction, causing increased intestinal permeability [28]. Therefore, through the intestinal mucosa repair in ALF can reduce the bacteria, LPS, or macromolecules pass through the tight connection into the body circulation and decrease the release of inflammatory factors within the liver, reducing the “second strike” for the liver.

In our cell experiment, we assessed that the IC50 concentration for NCM460 was 315.7 nM, which was much higher than our selective inhibitive concentration 120 pM for HDAC2. Therefore, we chose to treat cells with CAY10683 at 120 pM for 24 h in this experiment. To confirm the effects of CAY10683 on HDAC2 and histone, we firstly verify the effect of CAY10683 on HDAC2 and acetylation of histone H3. Stimulated by LPS, the expression of HDAC2 was increased in NCM460 cells. As expected, CAY10683 showed inhibition on HDAC2 in cells. The acetylated histone H3 was also promoted by LPS simulation and then enhanced by CAY10683, whereas CAY10683 could effectively decrease the permeability in LPS-stimulated NCM460 cell. The ZO-1 and occludin mRNA and protein levels were significantly decreased in the LPS-stimulated group, which was consistent with the reported study [29] and then increased after being treated with CAY10683. Next, we evaluated the effect of CAY10683 on the TLR4/MyD88 pathway in LPS-stimulated NCM460 cells. The TLR4 mRNA and TLR4, MyD88, TRIF, and TRAF6 protein levels were dramatically decreased after CAY10683 administration for LPS-stimulated NCM460 cells. In our animal experiment, we firstly insured that the ALF rat model was successfully established by D-galactosamine/LPS and then detected alleviating effect of CAY10683 on liver pathological changes and serum biochemical indicators in ALF rats. The histology and intestinal permeability changes of jejunum could be dramatically improved by CAY10683 in ALF rat model. Compared with model rat, the expression of HDAC2 was increased by CAY10683, and CAY10683 showed inhibition on HDAC2 ALF rats. The acetylated histone H3 was also promoted in the model group and then enhanced by CAY10683. To explore the specific molecular mechanism of protecting intestinal mucosal barrier, we detected that the ZO-1 and occludin mRNA and protein levels in the CAY10683 group were significantly elevated. Finally, we verified the protective effect of CAY10683 on the TLR4/MyD88 pathway in ALF rats.

Although the broad spectrum HDAC inhibitor TSA was shown to have the protective effect on intestine in ALF [6], which specific HDAC molecule related with the protective effect and the molecular mechanism of how HDAC2 inhibitor alleviated intestinal mucosal barrier in ALF were still unknown. Being an inhibitor of HDAC2, CAY10683 reduced the high expression of HDAC2 in ALF model. In this experiment, we found that the acetylated histone H3 increased under the stimulation by LPS. In the CAY10683 group, the acetylation of histones was further enhanced with HDAC2 reduction. The changes of HDAC2 and histone responding to CAY10683 in ALF model were similar to those in our previous studies [6, 30]. Because HDACs could not only regulate histones but also nonhistone proteins with multiple lysine residues acetylation, the gene expression or protein transcription could be upregulated or downregulated [31]. Moreover, the ubiquitinated or methylated modifications could participate in the protein regulation [32]. Therefore, the inflammatory pathway TLR4/MyD88 was suppressed, although the acetylated histone3 was increased in the CAY10683 group. The inflammatory pathway TLR4/MyD88 inhibition of CAY10683 was likely through nonhistone acetylation, and this acetylation process was through the effect of histone acetylation.

In conclusion, CAY10683 improved liver and small intestine histology, intestine permeability, and liver function in ALF rats. The inhibition of CAY10683 on inflammation is mainly via the histones acetylation. The present study not only proved that CAY10683 protected the liver in ALF rats as we previously described in ACLF rats but also further demonstrated its protection on intestine permeability. We speculated that CAY10683 could protect intestinal epithelial barrier disruption and keep the integrity of tight junction through inhabiting TLR4/MyD88 signal pathway, and CAY10683 can be considered as a therapeutic drug for protecting intestinal mucosa in ALF. This experiment not only focuses on the reliable curative effects but also provides a new avenue for clinical research and treatment and forming the foundation for future precision medicine for intestinal mucosal barrier in ALF. Further studies on the specific mechanism of CAY10683 regarding alleviating ALF may provide a potential prevention and treatment method for intestinal defense in ALF.

## Figures and Tables

**Table 1:** The primer sequence for RT-PCR.

Gene	Primer sequence (5′→3′)	
TLR4	Forward	TGACAGGAAACCCTATCCAGAGTT
(Human)	Reverse	TCTCCACAGCCACCAGATTCT
TLR4	Forward	TACAGTTCGTCATGCTTTCTC
(Rat)	Reverse	ATTAGGAAGTACCTCTATGCAG
ZO-1	Forward	GCAGCCACAACCAATTCATAG
(Human)	Reverse	GCAGACGATGTTCATAGTTTC
ZO-1	Forward	GCTCACCAGGGTCAAAATGT
(Rat)	Reverse	GGCTTAAAGCTGGCAGTGTC
Occludin	Forward	ACCCCCATCTGACTATGTGGAA
(Human)	Reverse	AGGAACCGGCGTGGATTTA
Occludin	Forward	TTACGGCTATGGAGGGTACAC
(Rat)	Reverse	GACGCTGGTAACAAAGATCAC
GAPDH	Forward	ACCACAGTCCATGCCATCAC
(Human)	Reverse	TCCACCACCCTGTTGCTGTA
GAPDH	Forward	GGCACAGTCAAGGCTGAGAATG
(Rat)	Reverse	ATGGTGGTGAAGACGCCAGTA
